# Non-coding repeat analyses in patients with Parkinson’s disease

**DOI:** 10.3389/fneur.2025.1606305

**Published:** 2025-07-22

**Authors:** Makito Hirano, Makoto Samukawa, Satoko Miyatake, Yuko Yamagishi, Chiharu Isono, Rino Yoshikawa, Kazumasa Saigoh, Atsushi Terayama, Yuji Higashimoto, Eriko Koshimizu, Takeshi Mizuguchi, Kanako Fujii, Yoshiyuki Mitsui, Naomichi Matsumoto, Yoshitaka Nagai

**Affiliations:** ^1^Department of Neurology, Kindai University Faculty of Medicine, Osaka, Japan; ^2^Department of Human Genetics, Yokohama City University Graduate School of Medicine, Yokohama, Japan; ^3^Department of Clinical Genetics, Yokohama City University Hospital, Yokohama, Japan; ^4^Division of Rehabilitation Medicine, Kindai University Hospital, Osakasayama, Japan; ^5^Department of Rehabilitation Medicine, Kindai University Faculty of Medicine, Osakasayama, Japan; ^6^Department of Rare Disease Genomics, Yokohama City University Hospital, Yokohama, Japan

**Keywords:** spinocerebellar ataxia type 8, repeat disease, parkinsonism, Canvas, *RFC1*, dysphagia, videofluoroscopic analysis

## Abstract

**Introduction:**

The genetic etiology of Parkinson’s disease (PD) is complex; approximately 10% of patients with PD have various gene mutations that lead to familial forms of the disease. Recent analyses of non-coding repeat regions revealed that many neurodegenerative diseases are associated with pathological expansions. We evaluated the genetic background of non-coding repeat expansions in Japanese patients with PD.

**Methods:**

We collected blood samples from 203 Japanese patients with PD and analyzed various non-coding repeat genes, including *ATXN8OS, RFC1, C9ORF72*, *NOTCH2NLC, BEAN1/TK2,* and *NOP56*, using PCR-Sanger sequencing, repeat-primed PCR assay, and long-read sequencing.

**Results:**

Three patients with PD (1.5%) were found to have heterozygous repeat expansions in *ATXN8OS,* the gene causative of spinocerebellar ataxia type 8 and is associated with long non-coding RNA. One (0.5%) patient had compound heterozygous repeat expansions (AAGGG and ACAGG) in *RFC1*, the gene causative of cerebellar ataxia, neuropathy, and vestibular areflexia syndrome, which encodes a DNA repair protein. No patient had repeat expansions in *C9ORF72, NOTCH2NLC, BEAN1/TK2,* or *NOP56*. All patients with *ATXN8OS* repeat expansions exhibited typical parkinsonism with relatively rare subjective dysphagia, which was confirmed by videofluoroscopic results. Functional imaging, such as dopamine-transporter single photon emission computed tomography, showed abnormal findings in patients with non-coding repeat expansions.

**Discussion:**

Our findings revealed the importance of non-coding repeat expansions in Japanese patients with PD. This is the first study to show the positive result of non-coding repeat expansions in many patients with PD in Japan.

## Introduction

Parkinson’s disease (PD) is clinically characterized by tremors, rigidity, and akinesia, which are later accompanied by postural instability. Radiological features were normal on conventional brain MRI but abnormal on dopamine transporter single-photon emission computed tomography or ^123^I-metaiodobenzylguanidine myocardial (MIBG) scintigraphy (1, 2). Dopamine replacement therapy is effective, at least in early-stage patients, but motor and non-motor complications, including reduced swallowing functions, become apparent in advanced stages. The progression, variation of symptoms, and response to treatment differ considerably between patients, suggesting that PD is associates with various implicated pathophysiological pathways (1). While PD mostly occurs sporadically, approximately 10% of patients with PD in Japan, as well as in Western countries, exhibit mutations in various genes that are responsible for familial PD. In addition, non-coding repeat expansions in genes causative of other neurodegenerative diseases have been reported worldwide in familial or sporadic PD. In contrast, no such findings have been established in Japan.

Spinocerebellar ataxia type 8 (SCA8) is an autosomal dominant neurodegenerative disease caused by non-coding CTA/CTG repeat expansions in *ATXN8OS* (ataxin 8 opposite strand). *ATXN8OS* is associated with long non-coding RNA, which, if not mutated, may not encode a functional protein (3, 4). The pathogenicity of the expanded allele has been proven using a transgenic mouse model (5). Many patients have pure cerebellar ataxia, whereas others have parkinsonism (6–8) or amyotrophic lateral sclerosis (9). Our preliminary analysis of 76 Japanese patients with PD did not reveal any *ATXN8OS* repeat expansions (8). To date, clinical data of only five PD patients exhibiting *ATXN8OS* repeat expansions have been reported globally, and none of these cases included imaging findings.

Cerebellar ataxia, neuropathy, and vestibular areflexia syndrome (CANVAS) have recently been attributed to biallelic non-coding pentanucleotide repeat expansions in *RFC1* (replication factor C subunit 1) (10). Normal *RFC1* encodes a DNA repair protein, the function of which is not altered by intronic repeat expansion (10). Pathogenic repeat configurations included AAGGG, ACAGG, AGGGC, AGAGG, and AAGGC (11). Recently, the disease entity has expanded to atypical phenotypes, including chronic or immune-mediated neuropathy without cerebellar ataxia or vestibular areflexia (12, 13). More recently, two reports from Northern Europe described that biallelic AAGGG repeat expansions are found in six patients with typical or early-onset PD (14, 15). A recent report from the US described that three patients with PD had presumably pathogenic biallelic AAGGG repeat expansions (16). Thus, *RFC1* mutations were likely causative in 11 patients with PD, most of whom had biallelic AAGGG repeat expansions, with the exception of complex types of AGGGG and AAGGG repeats (17).

Other non-coding repeat genes associated with parkinsonism include *C9ORF72* (Chromosome 9 open reading frame 72), causative for amyotrophic lateral sclerosis (ALS); *NOTCH2NLC* (notch 2 N-terminal like C) for neuronal intranuclear inclusion disease (NIID); *BEAN1* (*brain expressed associated with NEDD4 1*)*/TK2* (thymidine kinase 2) for spinocerebellar ataxia type 31 (SCA31); and *NOP56* (nucleolar protein 56) for spinocerebellar ataxia type 36 (SCA36). In cohorts of Western countries, 1.1% of patients with PD had GGGGCC repeat expansions in *C9ORF72* (18). Less than 1% of PD patients in China had *NOTCH2NLC* GCC repeat expansion (19). Several patients with ataxic *BEAN1/TK2* repeat expansions also presented with parkinsonism; however, they did not fulfill the criteria for a diagnosis of PD (20). Patients with GGCCTG repeat expansions in *NOP56* have abnormal dopamine transporter imaging findings consistent with those in patients with typical PD but lack clinical manifestation of parkinsonism (21).

The suppression of repeat expansion-associated toxicity and the removal of repeats are being actively investigated as a therapy for non-coding repeat diseases, such as *C9ORF72*-related ALS (22, 23). If such a therapy is established, it might be applicable to PD with non-coding repeats. In this report, we therefore analyzed non-coding repeat genes including *ATXN8OS*, *RFC1, C9ORF72*, *NOTCH2NLC*, *BEAN1/TK2,* and *NOP56* in 203 Japanese patients with PD.

## Materials and methods

### Genetic testing

All patients and controls were Japanese and enrolled in this study from the Kinki region in Japan between 2005 and 2024. All patients were diagnosed with PD by board-certified neurologists in accordance with the United Kingdom Parkinson’s Disease Society Brain Bank Clinical Diagnostic Criteria and had a Hoehn–Yahr grade of II–IV. A total of 203 patients with PD were included in the study, comprising 87 men and 116 women. The average age of the participants was 72 ± 11 years (mean ± SD). A control group consisting of 200 apparently healthy controls (116 men and 84 women; mean age ± SD, 71 ± 7 years) was also studied. Blood samples were collected from patients with PD who had no mutations in the *ATXN1, ATXN2, or ATXN3* genes (24–27). DNA was extracted using a DNA extraction kit or Pure Gene Blood Core Kit (Qiagen Inc., Germantown, MD, United States). The region containing the CTA/CTG repeat of the *ATXN8OS* gene was amplified using PCR with primers as described (3, 28). The amplified products were purified using gel electrophoresis and subjected to Sanger sequencing. The normal number of CTA/CTG repeats in the *ATXN8OS* gene ranges from 15 to 50, while repeats of length 80 or more are pathogenic. In several reports, expansions of more than 50 CTA/CTG repeats, including intermediate expansions, were stated to cause ataxia at some point in life; however, there were no clinical details. *BEAN1/TK2* was analyzed as described. *C9ORF72* and *NOP56* were analyzed by repeat-primed PCR assay as described (13, 27, 29). Primers used for the amplification of the short range of the repeat region in *RFC1* were as previously described (13). When no normal size band was detected, the sample was subjected to repeat-primed polymerase chain reaction (PCR) for AAGGG (pathogenic), ACAGG (pathogenic), AGGGC (pathogenic), AGAGG (possibly pathogenic), AAGGC (possibly pathogenic), AAAGG (variable penetrance), AAAAG (likely non-pathogenic), AAAGGG (likely non-pathogenic), and AAGAG (likely non-pathogenic) repeat configurations. The primers used for repeat-primed PCR are described by Hirano et al. (13). Long-read sequencing was performed using the Revio system (PacBio, Menlo Park, CA) following the manufacturer’s protocol. To increase the depth of coverage, hifi_reads.bam and fail_reads.bam, which did not pass the HiFi Q20 threshold, were merged and aligned using pbmm2.^1^ Genotyping and visualization of repeats were performed using TRGT v0.9.0 and TRVZ v0.9.0.^2^ We performed exome sequencing in some patients with pathological expansions using a previously described method (30). We focused on the known familial PD genes and then extended the search to other possible genes in which mutations resided, as listed in the Supplementary Table S2.

### Standard protocol approvals, registrations, and patient consents

This study was approved by the Institutional Review Boards of Kindai University (IRB# 16–011) and Yokohama City University (B230600048-Revised). All participants provided written informed consent to publish their clinical data.

### Evaluation of parkinsonism

Parkinsonism was evaluated in mutation-positive patients using part III (motor examination) of the Unified PD Rating Scale (UPDRS-III). All patients with *ATXN8OS* mutations were evaluated before and after dopaminergic treatment during “on” time.

## Results

### Results of genetic testing of PCR and sequencing analyses

**Figure 1:**
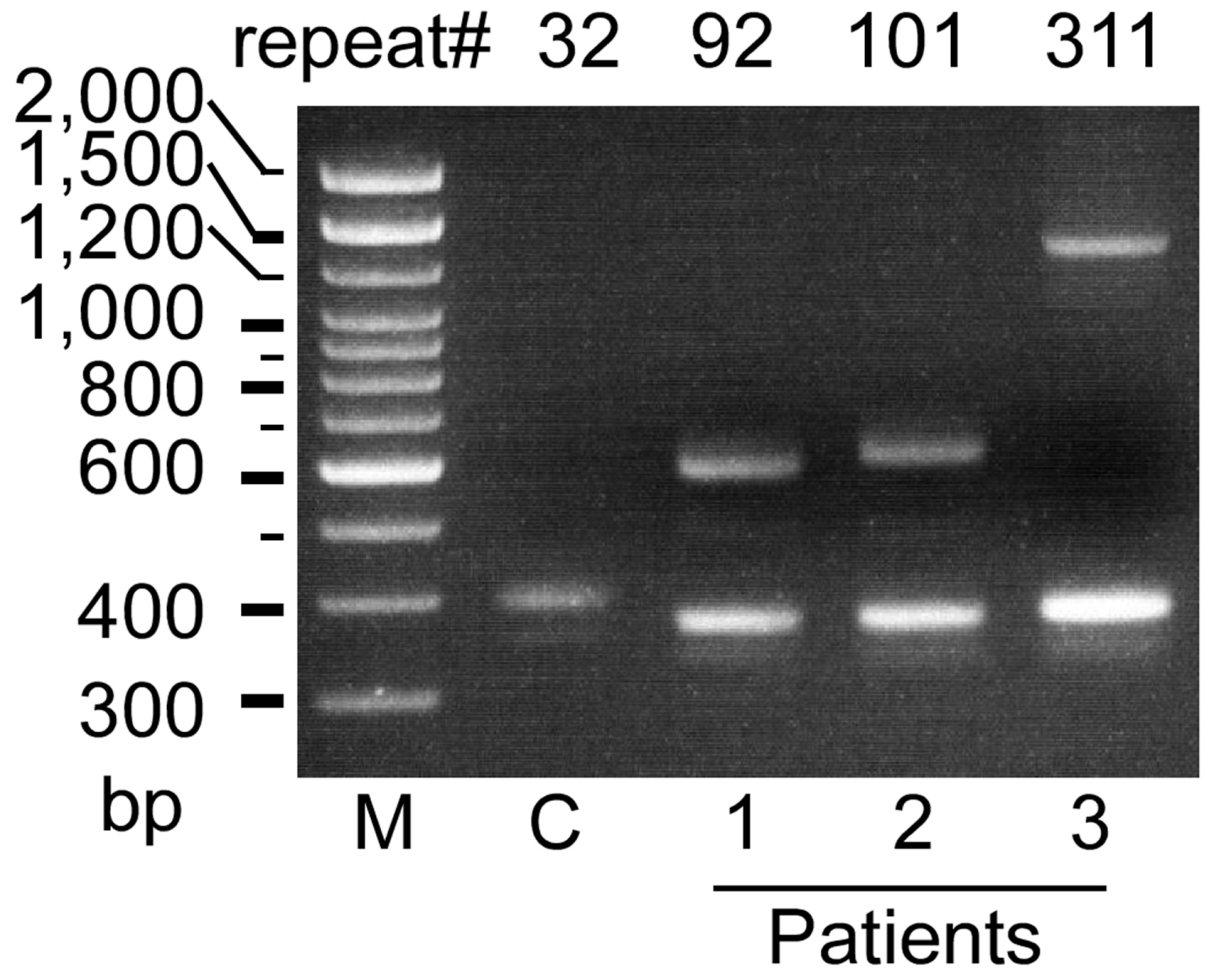
Agarose gel electrophoretic analysis of the *ATXN8OS* gene in patients with Parkinson’s disease (Pt 1–3). Three patients had expansions of the CTA/CTG repeat as indicated, whereas controls (C) had a normal repeat size. M, 100 base pair (bp) marker.

**Figure 2:**
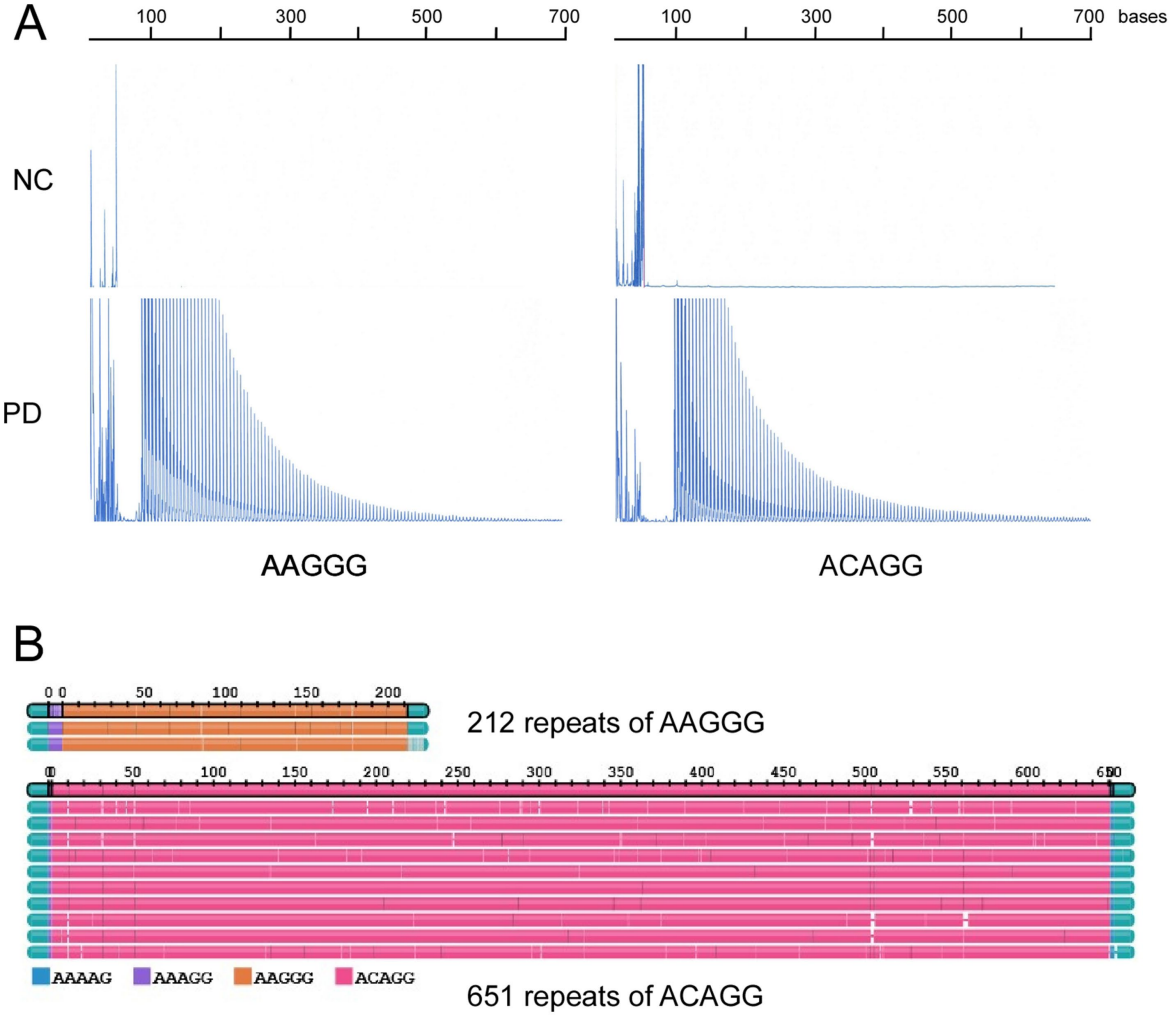
**(A)** Repeat-primed PCR results for *RFC1*. Pathological repeats of AAGGG or ACAGG were expanded in a patient with Parkinson’s disease (PD). **(B)** Long-read sequencing revealed 212 repeats of AAGGG and 651 repeats of ACAGG in the patient with PD.

Genetic testing revealed that three patients with PD had heterozygous *ATXN8OS* mutations (Figure 1). Short-range PCR for *RFC1* failed to identify a normal-size allele in one patient, and repeat-primed PCR revealed that the patient had compound heterozygous repeat expansions, AAGGG repeat and ACAGG repeat, in *RFC1* (Figure 2A). No other genes were mutated. None of the controls had mutations in the genes tested in this study. The control group had 26 ± 4 repeats (mean ± SD), ranging from 18 to 32, in the *ATXN8OS* gene. Patient 1 had 92 repeats (CTA13CTG1CTA1CTG77)_,_ Patient 2 had 101 repeats (CAT7CTG94), and Patient 3 had 311 repeats (CTA13CTG298).

### Results of long-read sequencing

Long-read sequencing revealed that the patient with *RFC1* had compound heterozygosity for 212 repeats of AAGGG and 651 repeats of ACAGG (Figure 2B). The pathogenic repeat length was originally described to be more than 400 (10), but several reports described that more than 100 were pathogenic (14, 15, 31).

### Results of exome analyses

We performed exome sequencing on one of the three patients with *ATXN8OS* expansions (Patient 3) and the patient with biallelic *RFC1* expansions. No pathological mutation was detected in either patient.

### Clinical information about patients with *ATXN8OS* mutations

**Figure 3:**
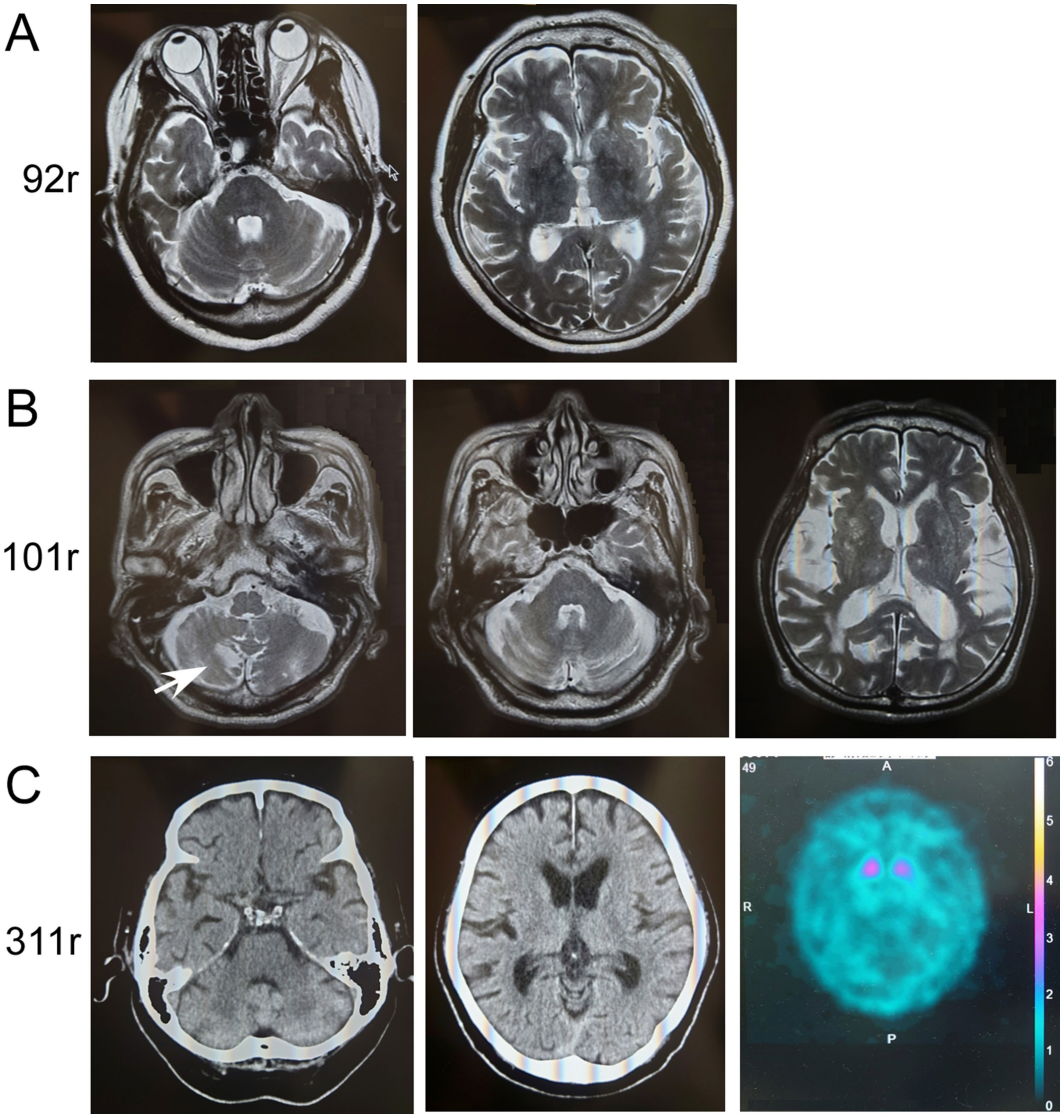
Imaging results of patients with *ATXN8OS* repeat expansions. **(A)** MRI of Patient 1 with 92 repeats (92r) showed no apparent atrophy of the cerebellum (upper panel) or cerebrum (lower panel). **(B)** MRI of Patient 2 with 101 repeats (101r) showed asymptomatic infarction of the cerebellum (arrow) and slight non-specific atrophy of the cerebellum or mild atrophy of the cerebrum with chronic ischemic lesions. **(C)** Computed tomography (CT) of the head in Patient 3 with 311 repeats (311r) showed no apparent atrophy of the cerebellum (left panel) or cerebrum (right panel). Dopamine-transporter single photon emission computed tomography showed marked reduction of the uptakes in the striatum. The specific binding ratio was 1.26 on the right striatum and 1.62 on the left.

**Figure 4:**
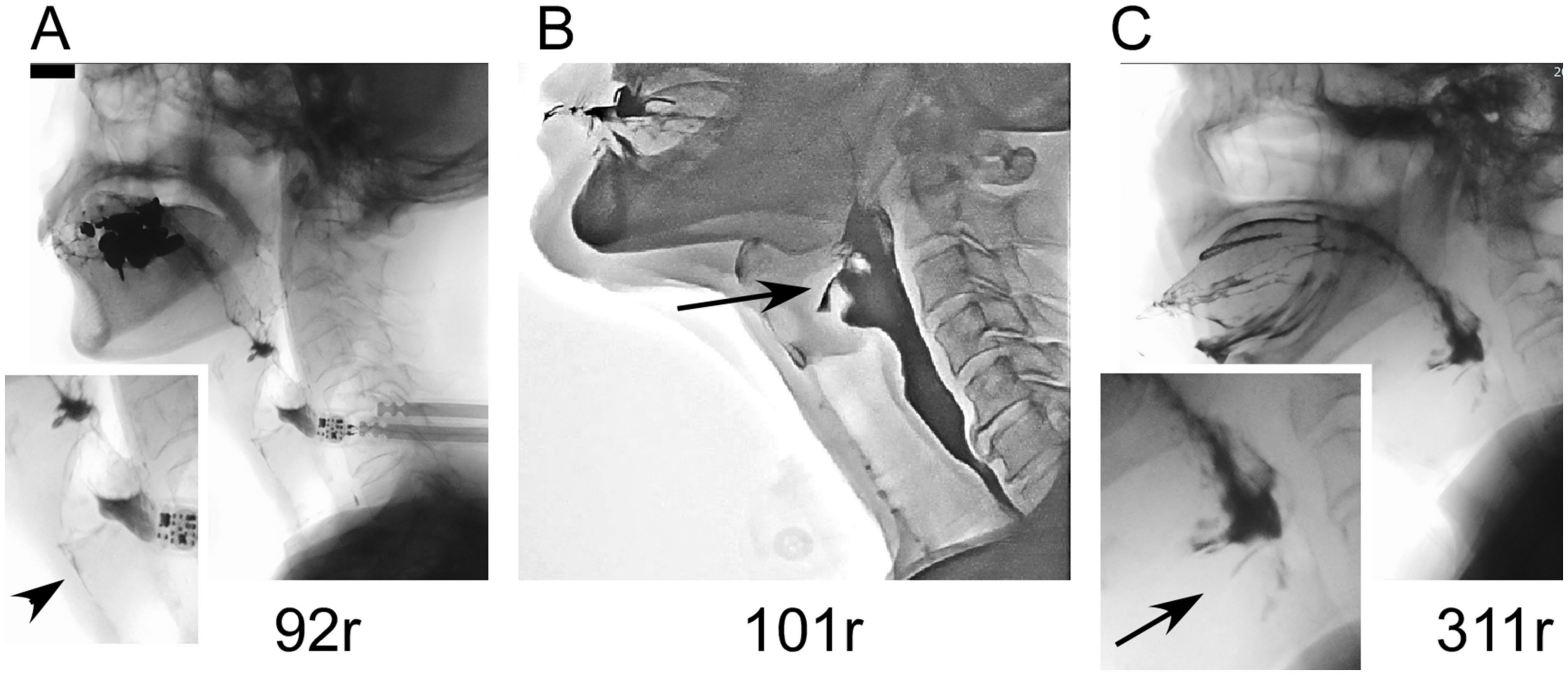
Videofluoroscopic results in patients with *ATXN8OS* repeat expansions. **(A)** A patient with 92 repeats underwent aspiration (arrowhead). **(B)** A patient with 101 repeats had laryngeal penetration. **(C)** A patient with 311 repeats also had laryngeal penetration.

The clinical information of the three patients with *ATXN8OS* mutations in this study, along with previously reported patients, is summarized in Table 1 (14, 15). Our patients responded well to levodopa or the dopamine agonist rotigotine. They seemed to have typical PD symptoms. According to the reported study (32), the phenotypes of our patients were as follows: Patients 1 and 3 were akinetic-rigid type, and Patient 2 was mixed type (Table 1). No patient had cerebellar ataxia. Imaging findings of the three patients with *ATXN8OS* mutations are shown in Figure 3. Patient 1, with 92 repeats, had no obvious abnormality on MRI. Patient 2 with 101 repeats had an asymptomatic cerebellar infarction with non-specific atrophy in the cerebrum and cerebellum (Figure 3B). Patient 3, with 311 repeats in *ATXN8OS,* had no obvious abnormality on MRI but showed reduced striatal dopaminergic transporter uptake (Figure 3C). Other patients did not undergo dopaminergic transporter imaging or other functional imaging studies. All patients with *ATXN8OS* mutations reported swallowing difficulties or discomfort in the throat after eating, which was supported by videofluoroscopic results (Figure 4). Patient 1 exhibited aspiration (Figure 4A), and Patients 2 and 3 exhibited laryngeal penetration (Figs. 4B,C). No correlation was found between the number of repeats and severity or age at onset because the sample size was too small for statistical analysis.

**Table 1:** Clinical information about PD patients with SCA8 mutations.

Patient#	1	2	3	4	5	6	7	8
Nationality	Japan	Japan	Japan	Taiwan	Taiwan	Taiwan	Taiwan	Korea
Family history	−	−	−	−	−	−	−	+
Sex	F	M	M	F	F	F	F	M
Age at examination	79	83	81	73	81	71	58	49
Age at onset	67	77	74	60	71	67	57	43
Phenotype	Akinetic-rigid	Mixed	Akinetic-rigid	nd	nd	nd	nd	nd
Tremor at rest	- - > +*	+	+	+	+	+	+	nd
Rigidity	+	+	+	+	+	+	+	+
Bradykinesia	+	+	+	+	+	+	+	+
Dysphagia (videofluorography)	Aspiration	Laryngeal penetration	Laryngeal penetration	nd	nd	nd	nd	nd
Levodopa responsiveness	+	+	+	+	+	+	+ (dopamine replacement therapy)	+ (DA agonist)
UPDRS III (pre/post treatment)	13- > 9 (levodopa 200 mg)	18- > 12 (Rotigotine patch 2 mg = LED 60 mg)	38- > 35 (L-dopa 200 mg) - > 20 (L-dopa 300 mg)	nd	nd	nd	nd	29- > 14
Repeat #	92	101	311	88	75	82	92	103
Context	CTA13 CTG1 CTA1 CTG77	CTA7 CTG94	CTA13 CTG298	CTA8 CCA1 CTA1 CTG1 CTA1 CTG1 CTA1 CTG74	CTA20 CTG2 CTC1 CTG52	CTA12 CTG70	CTA7 CTG2 CTA1 CTG1 CTA1 CTG80	nd
Note		Asymptomatic cerebellar infarction		Motor fluctuations	Motor fluctuations			Dysmetria in the bil hands, dystonic posture in the right arm at the age of 51
References	This report	This report	This report	Clin Genet 2004;65:209.	Clin Genet 2004;65:209.	Clin Genet 2004;65:209.	Clin Genet 2004;65:209.	J Clin Neurol 9;274:2013

PD, Parkinson’s disease; *Tremor was not apparent at the first examination but later developed; nd, not described; bil, bilateral.

### Clinical information about patients with *RFC1* mutations

**Figure 5:**
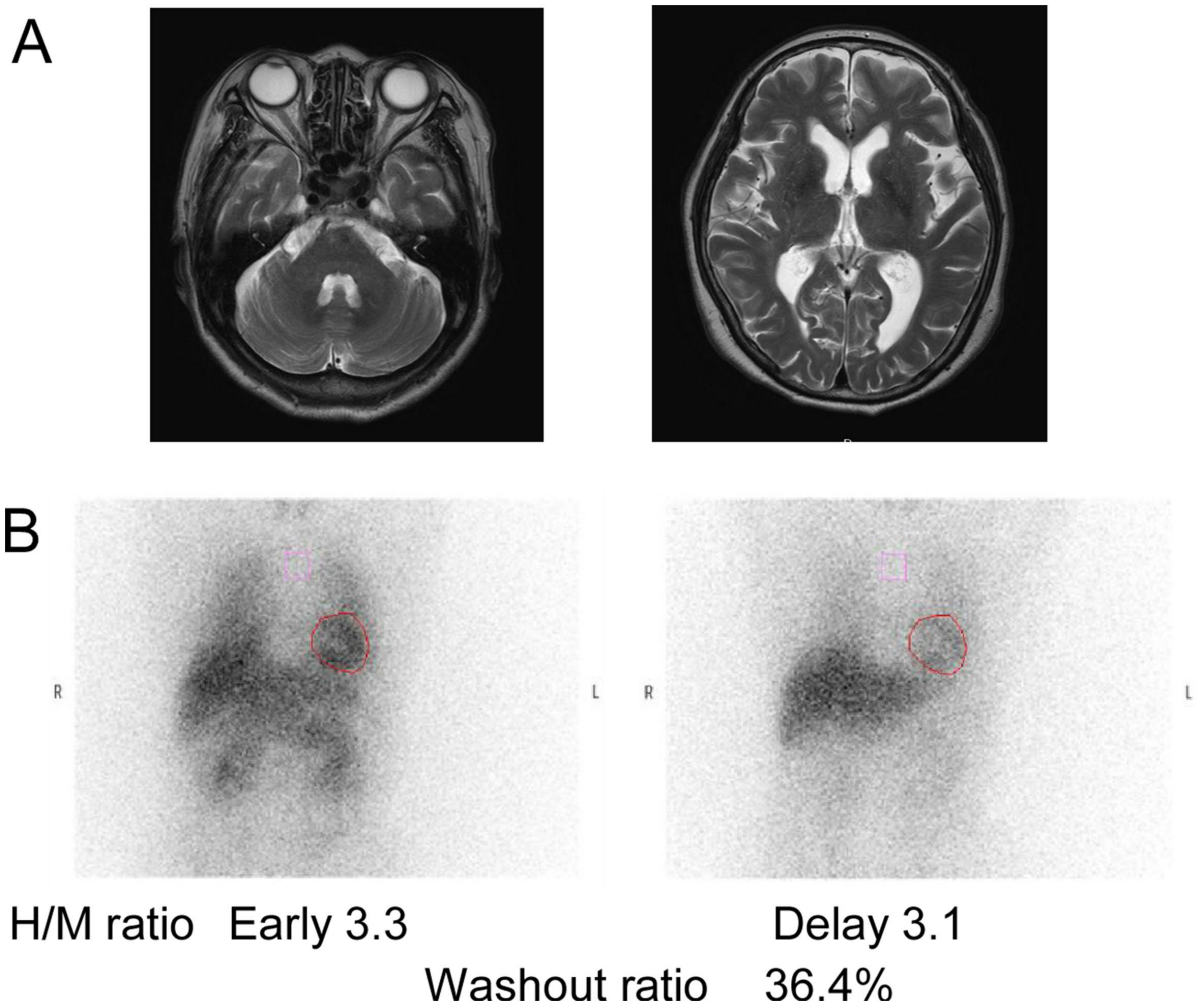
Imaging results of patients with *RFC1* repeat expansions. **(A)** MRI of the patient with AAGGG and ACAGG repeat expansions. **(B)** ^123^I-metaiodobenzylguanidine myocardial scintigraphy performed on a patient about 1 year after onset showed normal values of heart/mediastinum (H/M) ratio. The H/M ratios were standardized under medium-energy collimator conditions (normal ≥ 2.2). Additionally, the washout ratio was found to have increased (normal ≤ 22%).

The clinical information of one patient with *RFC1* mutations, along with previously reported patients, is summarized in Table 2 (15, 16). The patient had no cerebellar ataxia. Our patient had the oldest age of onset for the disease but had a typical akinetic-rigid phenotype. One of the four patients with biallelic AAGGG repeats reported in the US also had an additional *LRRK2* mutation. This suggests that in this patient, the PD could have been caused by the *LRRK2* mutation and not by the *RFC1* repeat expansion and therefore was excluded from the table in this study. Imaging results of a patient with *RFC1* repeat expansions are shown in Figure 5. The patient underwent MIBG scintigraphy and was found to have a normal heart/mediastinum (H/M) ratio but an increased washout ratio about 1 year after onset. No patients with *RFC1* repeat expansions underwent videofluoroscopic analysis. No correlation was found between the number of repeats and age at onset because the sample size was too small for statistical analysis.

**Table 2:** Clinical information about PD patients with *RFC1* mutations.

Patient#	1	2	3	4	5	6	7	8	9	10	11	12
Nationality	Japan	Finland	Finland	Finland	Finland	Finland	Finland	USA	USA	USA	China	China
Sex	F	M	M	F	M	F	F	F	F	F	M	M
Age at examination	82	73	69	64	74	61	69	58	66	55	52	48
Age at onset	74	65	59	51	47	40	48	58	66	55	44	33
Phenotype	Akinetic-rigid	Akinetic-rigid	Tremor-dominant	Tremor-dominant	Tremor-dominant	Tremor-dominant	Akinetic-rigid	nd	nd	nd	nd	nd
MMSE	nd	20	30	nd	8	nd	29	23 (MOCA)	29 (MOCA)	29 (MOCA)	27	27
Hallucination	−	+	+	−	nd	nd	nd	nd	nd	nd	nd	nd
RBD	nd	+	+	−	nd	nd	nd	−	+	−	nd	nd
OH	−	++	++	−	+	−	−				nd	nd
DTR	Reduced	Absent	Brisk knee jerks	Normal	nd	nd	nd	nd	nd	nd	nd	nd
Repeat configuration	AAGGG	AAGGG	AAGGG	AAGGG	AAGGG	AAGGG	AAGGG	AAGGG	AAGGG	AAGGG	AGGGGexp (AAGGG14)	AAGGG*
	ACAGG	AAGGG	AAGGG	AAGGG	AAGGG	AAGGG	AAGGG	AAGGG	AAGGG	AAGGG	AAGGG*	AAGGG*
Repeat number	212	144	720	643	311	141	228	360	599	403	119	600
	651	765	812	820	321	410	831	866	1,183	648	1,000	750
Family history	−	−	−	−	−	+	−	+	−	−	−	−
References	This report	npj Parkin Dis 2022;8:6	npj Parkin Dis 2022;8:6	npj Parkin Dis 2022;8:6	Eur J Neurol. 2023;30: 1,256	Eur J Neurol. 2023;30: 1,256	Eur J Neurol. 2023;30: 1,256	npj Parkin Dis 2024;10: 108	npj Parkin Dis 2024;10: 108	npj Parkin Dis 2024;10: 108	npk Parkin Dis 2025;11:1	npk Parkin Dis 2025; 11:1

MOCA, Montreal cognitive assessment; *AATGG expansion was inserted as a possible somatic mutation; ex, expansion.

## Discussion

This study found that three patients with PD (1.5%) had heterozygous mutations in *ATXN8OS* and that one patient with PD (0.5%) had compound heterozygous mutations in *RFC1*. In contrast, no patient had a mutation in *C9ORF72, NOTCH2NLC, BEAN1/TK2*, or *NOP56,* some of which have been mutated in PD in other countries (18, 33). These findings suggest that pathogenic non-coding repeat expansions are occasionally associated with PD in Japan, with some racial or geographical differences. Especially, *ATXN8OS* mutations were more frequently found in patients with PD and related disorders in East Asia (6, 8, 34).

Interestingly, the three patients with *ATXN8OS* mutations reported subjective dysphagia, which was confirmed by the objective examination using videofluoroscopic analysis. Patients with PD without mutations also frequently have dysphagia, but only 39% of patients reported subjective dysphagia in our previous cohort (35). The swallowing disturbance found in *ATXN8OS* mutation-positive patients is reminiscent of our previous finding in *ATXN8OS*-related ALS, where three of the three mutation-positive patients had bulbar onset or rapid progression of dysphagia (9). We speculate that neurons associated with swallowing may be susceptible to the expanded repeats in *ATXN8OS.*

The role of expanding non-coding repeats in the etiology of PD remains unclear, but a recent pathological study on SCA8 revealed that all four patients had degeneration of the substantia nigra, which is a region that is also affected in PD. Tau pathology typical of supranuclear palsy (PSP) was found in only one patient (36), suggesting an unknown mechanism involved in the degeneration. In addition, a pathological study on a patient with CANVAS revealed depletion of the pars compacta of the substantia nigra with widespread Lewy bodies in the locus coeruleus and substantia nigra, regions affected in PD (37). The tendency for substantia nigra degeneration in such non-coding repeat diseases may be associated with striatal dopamine deficiency. Although the effect of non-coding repeat expansion on existing PD treatments is unknown, PD patients with non-coding repeats in the current and reported studies responded well to conventional dopamine replacement therapy, such as oral levodopa or dopamine agonists.

Imaging results, especially functional imaging, have been rarely reported in patients with non-coding repeat expansions. Dopamine-transporter single photon emission computed tomography in this study showed a marked reduction of striatal uptake, a cardinal feature of PD. MRI revealed no apparent atrophy of the cerebellum in the three patients with *ATXN8OS* mutations. This is compatible with the reported findings of *ATXN8OS*-related PD (6). Similarly, *RFC1*-related PD showed no apparent atrophy in the cerebellum. A patient with *RFC1* repeat expansions a year after onset had an apparently normal H/M ratio in MIBG scintigraphy, which was supposed to be reduced in PD. However, H/M ratios are sometimes within normal ranges during the early phase of PD (2). In contrast, the increased washout ratio observed here was compatible with that in PD (38). These findings suggest that imaging findings in PD associated with non-coding repeat expansions are indistinguishable from those without repeat expansions.

Non-coding repeat expansions may have some common pathomechanisms, including the formation of RNA foci and repeat-associated non-ATG (RAN) translation (39). In SCA8, both mechanisms have been reported to be involved (5, 40, 41). In addition, the mechanism underlying the loss of function of genes with non-coding repeats has attracted much attention (22). A therapeutic approach to *C9ORF72*-related ALS may also be applicable to other non-coding repeat diseases. In *C9ORF72*-related ALS, the suppression of abnormal transcription by antisense oligonucleotides is an ongoing clinical project (42). A similar method was recently reported in SCA36 (43). In another study, the suppression of toxicity in an abnormal *ATXN8OS* transcript by the KH RNA-binding domain of Spoonbill *in vivo* exerted a therapeutic effect (44). Recently, excision of pathological repeats has been reported in experimental models of *C9ORF72*-related ALS (22). Although the *ATXN8OS* gene, which is reported to have bidirectional transcripts, may have a more complex pathomechanism (5), the suppression of at least one pathological pathway might help slow the disease process.

The pathogenesis of *RFC1* repeat expansions remains to be elucidated, but recessive inheritance suggests the loss of function of *RFC1*. A patient with one truncated mutation in an allele and a repeat expansion in the other supports this notion. However, a loss-of-function mechanism of RFC1 has not been proven yet (10). A recent report showed RNA foci in the cerebellum, suggesting the dominant toxic function of the expanded repeat. The configuration of repeats has been shown to be crucial for pathogenicity in this gene, but of the pathogenic repeat configurations, AAGGG, ACAGG, AGGGC, or combinations thereof showed no clear genotype and phenotype relationship in total patients with CANVAS (11, 12, 45). Interestingly, our patient with PD had only ACAGG repeats and had the highest age of onset. The association between repeat configuration and age at PD onset should be assessed as more mutation-positive patients are identified. Thus, the pathogenesis of the *RFC1* repeat expansion may involve multiple pathways.

In this study, the coincidental occurrence of SCA8 and PD was possible because mutations in the aforementioned genes have been infrequently found in controls (3, 4, 46). However, the repeat sizes found in patients with PD, 92–311 repeats, were not found in the reported control alleles in Japan (*n* = 654) (46). The relatively low prevalence of SCA8 (0.7/100,000) and PD (1.8/1,000) in Japan suggests that their coincidental coexistence is unlikely to occur in the three presumably unrelated patients. In contrast, because *RFC1* mutations are infrequently found in controls, only one patient with PD may have coincident PD. Therefore, future studies on their correlation. In summary, this study reveals that a certain number of patients with PD were positive for non-coding repeat expansions and extends the geographic range of such patients to Japan. In the future, new therapies for non-coding repeat disease are expected to be developed for this disease group.

## Data Availability

The original contributions presented in the study are included in the article/Supplementary material, further inquiries can be directed to the corresponding author.

## Footnotes

^1^ https://github.com/PacificBiosciences/pbmm2

^2^ https://github.com/PacificBiosciences/trgt?tab=readme-ov-file
