# Supplementary material for: Non-coding repeat analyses in patients with Parkinson’s disease
Source: Front Neurol. 2025 Jul 22;16:1606305. doi: 10.3389/fneur.2025.1606305 (PMC12321559; doi:10.3389/fneur.2025.1606305)
Supplement: Supplementary file 1 [file Table_1.docx]

| Table S1. List of abbreviations used in this study | |
| --- | --- |
|  |  |
| Abbreviation | Definition |
| ALS | amyotrophic lateral sclerosis |
| ATXN8OS | ataxin 8 opposite strand |
| BEAN1 | brain expressed associated with NEDD4 1 |
| C9ORF72 | Chromosome 9 open reading frame 72 |
| CANVAS | Cerebellar ataxia, neuropathy, and vestibular areflexia syndrome |
| MIBG | ^123^I-metaiodobenzylguanidine myocardial |
| NIID | neuronal intranuclear inclusion disease |
| NOP56 | nucleolar protein 56 |
| NOTCH2NLC | notch 2 N-terminal like C |
| PD | Parkinson’s disease |
| RAN | repeat-associated non-ATG |
| RFC1 | replication factor C subunit 1 |
| SCA8 | Spinocerebellar ataxia type 8 |
| SCA31 | Spinocerebellar ataxia type 31 |
| SCA36 | spinocerebellar ataxia type 36 |
| TK2 | thymidine kinase 2 |
