# Supplementary material for: Non-coding repeat analyses in patients with Parkinson’s disease
Source: Front Neurol. 2025 Jul 22;16:1606305. doi: 10.3389/fneur.2025.1606305 (PMC12321559; doi:10.3389/fneur.2025.1606305)
Supplement: Supplementary file 2 [file Table_2.docx]

| Table　S2. Genes associated with Parkinson's disease | |
| --- | --- |
|  |  |
| Gene name | Transmission |
| *SNCA* | AD |
| *PRKN (PARK2)* | AR |
| *UCHL1* | AD |
| *PINK1* | AR |
| *DJ-1* | AR |
| *LRRK2* | AD |
| *ATP13A2* | AR |
| *GIGYF2* | AD |
| *HTRA2* | AD |
| *PLA2G6* | AR |
| *FBXO7* | AR |
| *VPS35* | AD |
| *EIF4G1* | AD |
| *DNAJC6* | AR |
| *SYNJ1* | AR |
| *CHCHD2* | AD |
| *VPS13C* | AR |
| *GBA* | AD |
| *PSAP* | AD |
| *PARK12* | AD |
| *MAPT* | AD |
| *PTPA* | AD |
| *ADHC1* | AD |
| *PARK21* | AD |
| *FLNA* | AD |
| *GCH1* | AD |
| *RFC1* | AR |
| *DNAJC13* | AD |
| *TMEM230* | AD |
| *RIC3* | AD |
| *SPG11* | AR |
| *PODXL* | AR |
| *PTRHD1* | AR |
| *RAB39B* | X-linked |
| AD, Autosomal dominant; AR, Autosomal recessive | |
